# The role of non-governmental organisations in the management of separated and unaccompanied children, following disasters in Iran

**DOI:** 10.1186/1756-0500-3-256

**Published:** 2010-10-07

**Authors:** Farnaz Bazeghi, Hamid R Baradaran

**Affiliations:** 1 The Nuffield Centre for International Health & Development, Leeds Institute of Health Sciences, University of Leeds, Leeds LS2 9LJ, UK; 2 Department of Epidemiology, Iran University of Medical Sciences, Tehran 14496, Iran; 3 Center for Nursing Care Research, Iran University of Medical Sciences, Tehran 14496, Iran

## Abstract

**Background:**

Following disasters, separated and unaccompanied children are among the most vulnerable, therefore international organisations have formed guidelines regarding the management of these children. Guidelines include recommendations for identifying and registering children, tracing family members, reunification and arrangements for interim and durable care. There is a lack of experiential evidence on how these principles are put into practice at operational levels, and whether existing policies were useful. There is a particular lack of empirical evidence from the disaster prone country of Iran. The aim of this study was to describe the role of Non- Governmental Organisations (NGOs) in the management of separated and unaccompanied children, following disasters in Iran in order to plan for and provision of future disasters.

**Findings:**

The Iranian Red Crescent Organisation, Committee Emdad Imam Khomeini (a national organisation unique to Iran that is protected by the government and supported by public contributions) and Behzisti (the government welfare organisation in Iran) are the main figures involved in the management of separated and unaccompanied children, following disasters in Iran. NGOs are rarely responsible for caring for unaccompanied children, however they provide valuable support including financial assistance, arrangement of educational and extra-curricular activities and psychosocial support. Following the initial chaos after the Bam earthquake, international guidelines on separated and unaccompanied children were largely followed.

**Conclusions:**

Systems for managing separated and unaccompanied children following disasters in Iran, involving NGOs, are emerging. However, most are yet to be formalised.

## Background

Disasters are inevitable, therefore disaster management warrants attention and is essential in disaster prone countries such as Iran [1-3]. Children who are unaccompanied, meaning they have do not have a responsible adult as their guardian, or separated from their usual care-givers are particularly vulnerable following disasters [4-9]. As disaster situations often breakdown existing social structures and services within a country [4], humanitarian organisations can provide valuable assistance. Therefore, such organisations have produced guidelines for the management of separated and unaccompanied children following disasters.

There is a lack of experiential evidence on the management of separated and unaccompanied children following disasters, evidence that would be valuable in planning for and provision of future disasters. This study aims to contribute to the small number of published empirical reports [3,10,11] regarding disaster management, by interviewing personnel from relevant Non-Governmental Organisations (NGOs) in Tehran and Bam, in Iran. The main aim of this paper is to describe the role of NGOs in the Management of Separated and Unaccompanied Children, following Disasters in Iran.

## Methods

### Study Region

Part one of the study took place in Iran's capital, Tehran. Many of the disaster management protocols in Iran are coordinated from Tehran. Part two of the study was conducted in the earthquake affected region of Bam; a suitable research site as many NGOs are still active there following the disaster in 2003.

### Study Design

Research was conducted within a six week period. A combination of 25 informal meetings and semi-structured interviews, that were in-depth when appropriate, were carried out. Interviews involved a mixture of open and closed questions, and were designed to last between 30 and 40 minutes. The interview was structured according to recommendations on qualitative research [12-14]. The content of each question was developed from the guidelines [1,2,4-7,15,16] examined as part of the literature review conducted prior to field work. Interviews took place in the organisation's headquarters, or in mutually convenient locations.

The following people were present at each interview:

1. Interviewer (Principal Investigator (FB): International Health BSc Student)

2. Interviewee (Subject from relevant organisation)

3. Male translator and chaperone

The translator served a dual role as a chaperone because the Principal Investigator (FB) is female and was unfamiliar with the country. The translator/chaperone was briefed prior to conducting research.

Additional literature was collected from organisational headquarters, or from recommended sources, throughout the study since literature relating to Iran is limited in the UK.

### Recruitment Strategy

An ideal and systematic recruitment strategy would have involved obtaining a comprehensive list of the NGOs contributing to the management of separated and unaccompanied children, following disasters in Iran. From this list each agency would be ranked in order of relevance, contacted and meetings arranged with as many relevant NGO personnel as possible. A more realistic attainment was to recruit a convenience sample; obtained by identifying suitable international, national and local organisations through personal contacts, journal articles, peer reviewed literature and via the World Wide Web. Once the relevant personnel within each organisation had been identified, meetings were provisionally arranged via email and phone, while the data regarding each NGO and their personnel was entered into a spreadsheet and ranked in order of relevance (table 1) The timing of interviews was to be confirmed once the principal investigator arrived in Iran.

**Table 1:** Organisations interviewed

Organisation		Meeting/Interview Number	Name of Organisation
Governmental		2	Ministry of Heath and Social Welfare
		13	Educational Research Centre of Behzisti (Iranian Welfare Organisation) Tehran
		14	Behzisti, Tehran
		15	Behzisti, Tehran
		17	Behzisti, Bam
		21	Ministry of Health and Medical Education for Bam
		22	Department of Construction in the Ministry of Health
		23	Family Reunification Project (Behzisti)
Organisation headed by Supreme Leader of Iran		3	Committee Emdad Imam Khomeini
**Non-Governmental**	**International**	18	Support to Life
	**National**	5	Iran University of Medical Sciences
		6	University of Social Welfare and Rehabilitation Sciences
		11	Khane Madarah va Koodak (Tehran)
		19	Moshtameh Tavenbakhshe Iman Reza
		20	Khane Madarah va Koodak (Bam)
		28	Association for the Protection of Child Labourers
	**Local**	4	Khane Sabze
		9	Zeinab Kobra Foundation
		10	SPASDI
		24	Bam Blue Crescent
		25	Bavarat Community Centre, Play scheme
		26	Khale Sara kindergarten
		27	Hazrat Fetemeh Centre
			
**Inter-Governmental United Nations**	**World Health Organisation (WHO)**	1	WHO: Health Systems Specialist
		7	WHO: Field worker (Bam)
			
	**United Nations Children's Fund (UNICEF)**	12	UNICEF: Programme Officer for Iran
		16	UNICEF: Assistant Project Officer Child Protection and Family Reunification
		23	Family Reunification Project (UNICEF)

Research began with a meeting with a Health Systems Specialist at the World Health Organisation (WHO) office in Tehran. Here the spreadsheet of contacts was reviewed, and advice obtained on the best manner in which to approach people. Further contacts were given, and most importantly the Principal Investigator was introduced to the Managing Director of Health Improvement at the Ministry of Health and Social Welfare, in Tehran. A meeting with the Managing Director of Health Improvement, involving an explanation of this study and presentation of the British ethical approval it received, was conducted. Following the meeting a letter acknowledging this study, that also had the dual role of introducing the Principal Investigator to other agencies, was prepared. A copy of this letter was also faxed to the Ministry of Health and Social Welfare in Bam, ahead of our arrival. This official letter was the most useful resource for this study in Iran, as all interviewees were comforted by the fact that this project had been approved by the Ministry of Health and Social Welfare, meaning that everyone approached was willing to be interviewed.

Initial discussions with academics in Iran revealed that the majority of work in the country is done through personal contacts. Therefore, meetings to build relationships and seek guidance became necessary. Through such meetings introductions were made to other subjects; these were vital in order for subjects to agree to being interviewed.

Prior to arrival in Iran, the interview questions were emailed to a WHO field worker on request. This subject took it upon themselves to interview three relevant NGOs in Bam and return the data to the Principal Investigator via email. In order to validate these results, a meeting with the WHO field worker was conducted where the results were discussed and clarified so they could be included in the study.

### Study Sample

Due to time constraints and the depth of the interviews and meetings being conducted, the sample size could not consist of more than 25 subjects. All subjects are, or have been, involved in the management of separated and/or unaccompanied children, following a disaster(s) in Iran. The sample size was sufficient to answer the research question and includes an adequate variety of opinions at the required depth. Limiting the sample size also ensured accurate translating and transcribing in the given time.

Meetings and interviews were not only with NGOs, as originally planned, because during initial meetings in Iran it became evident that it was necessary to also interview relevant personnel from other agencies, for the findings to be complete and accurate.

### Ethical Considerations

The information sheet relating to this study was translated into Persian (Farsi), and offered to subjects to allow them to give informed consent when opting to participate in this study. Confidentiality and anonymity were always respected, with only the name of each organisation and the interviewee's job title according to their own description being included. References to the subject's personal details from which they could be identified were also omitted. The fact that there would be no negative repercussions from participating in the study was stressed. The verbal consent was obtained as, considering the circumstances, this was deemed the most appropriate and ethical method.

The study design was ethically approved by the ethics board at the Nuffield Centre for International Health and Development, Leeds. Ethical Approval was not required by any ethics committees in Iran.

### Data Collection

Interviews and meetings were conducted in Persian (Farsi) language by the Principal Investigator, with the translator only assisting in maintaining interviews when necessary. Three interviews that were conducted in English, as the subjects were equally as confident in English as they were in Persian (Farsi). With informed consent from subjects, interviews were recorded to allow accurate transcription and the opportunity to refer to the exact content of the interview at a later date.

Ideally results would have been transcribed in Persian (Farsi) first and then translated into English. However, time constraints prevented this from being possible. Translation of interviews was done in Iran as translators were available to assist when required. Furthermore, translation and transcription in Iran provided the opportunity for any gaps in the research to be identified, and these could then be researched before leaving the country. Transcription was done word for word wherever possible; however the nature of language prevented literal translation in some instances. Copies of transcriptions can be obtained from the author on request. Following transcription all recordings were erased to ensure confidentiality and anonymity.

### Data Analysis

Use of the Grounded Theory Approach was suitable in this study [12-14] as was thematic analysis [12,13], in order to identify recurrent or common themes in the participant's responses. Following the principles of thematic analysis subheadings were created and the content of each transcript was analysed, and then put under the relevant subheading. Furthermore, the additional literature acquired in Iran was analysed along with the hand written notes made following each interview and throughout the research. Any relevant information from these sources was then also included under the appropriate subheadings.

## Results

**Figure 1:**
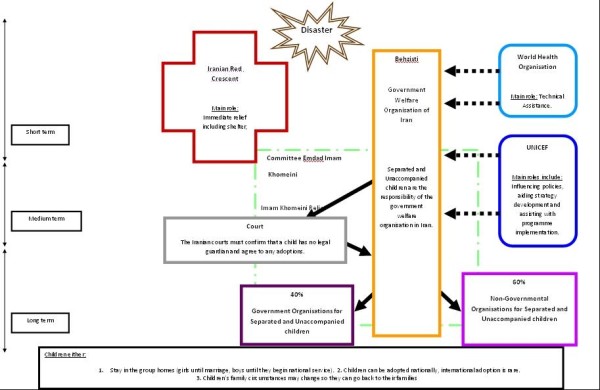
**Overview of the management of separated and unaccompanied children following disasters in Iran**.

**Figure 2:**
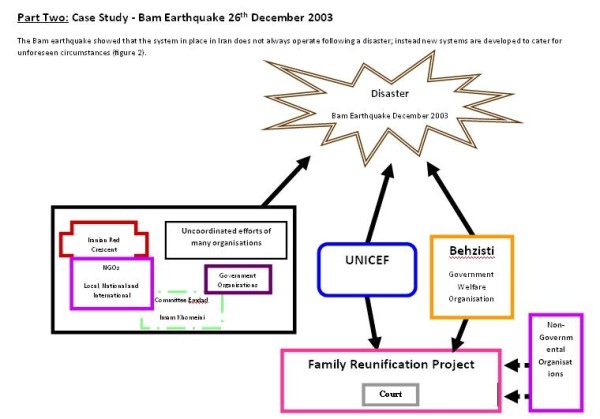
**management of separated and unaccompanied children following the Bam earthquake**.

Part one of this study relates to Figure 1, and gives an overview of the management of separated and unaccompanied children, following disasters in Iran. Part two relates to Figure 2, and will describe the role of NGOs specifically in the management of separated and unaccompanied children, following the Bam earthquake.

Part One: Overview of the Management of Separated and Unaccompanied Children, following Disasters in Iran

The three parties below are the main figures involved in the management of separated and unaccompanied children, following disasters in Iran:

1. Iranian Red Crescent Organisation

2. Committee Emdad Imam Khomeini National organisation unique to Iran that is protected by the government and supported by public contributions)

3. Behzisti (Government Welfare Organisation of Iran)

Despite the fact that some of the organisations included in this study are not strictly NGOs, most are still regarded as NGOs in the broader sense and all have central roles in the care of separated and unaccompanied children, following disasters in Iran. Therefore, the responsibilities of these agencies must be outlined in order to give a more complete picture of the management process, and put the role of NGOs in context.

### Behzisti (Government Welfare Organisation of Iran)

Behzisti is responsible for separated and unaccompanied children in Iran. However, these children are not necessarily separated or unaccompanied as a result of a disaster:

"Children come into the care of Behzisti because they have gotten lost, they have been abandoned in places like mosques, they have been found by the police ... legally all children in these circumstances should be brought to Behzisti" (Interview 14)

As a number of children are inevitably separated or unaccompanied following a disaster, Behzisti is active immediately after a disaster occurs and continues it's work into the long term phases of the management process:

*"Immediately after a disaster we [Behzisti] setup camps with psychiatrists, psychologists and social relief workers ..." (Interview 13)*

When a child has become separated from their family, for whatever reason, the main aim is to search for the child's family and reunite them, where possible and suitable:

"...after investigating the circumstances to see why the child was left...our main aim is to return these children to their families." (Interview 14)

When reunification is not an option children are placed in residential care either in the private (NGOs) or public sector:

"60% of the services for guardian-less children are provided in co-operation with the private sector, and 40% are carried out by the public sector." (Interview 15)

According to Behzisti, NGOs who are given children to care for have to uphold certain standards set by Behzisti. They also provide financial assistance and regular supervision:

" ..we do give these [guardian-less] children to NGOs, these foundations work under our supervision receiving financial and consultative assistance... an NGO who gets a child from us, if they don't maintain our standards, we take the child back from them..." (Interview 13)

"Standards relating to the building [where children are housed] also need to be upheld.. for every 8 children there should be at least one carer, and each centre should have at least three instructors, working on a shift bases so they change every 8 to 12 hours...each child should also have a private room, and there should be social spaces and spaces to study." (Interview 15)

### Committee Emdad Imam Khomeini

Committee Emdad Imam Khomeini, a national organisation unique to Iran, was established officially following the Islamic revolution. The organisation aims to:

"... bridge the gap between those who are wealthy and those who are not....we are protected by the government and we are supported by public contributions." (Interview 3)

One of Committee Emdad Imam Khomeini's specific duties is the "support of needy people and families against the problems coming from accidents and natural and non-natural disasters" [17].

Committee Emdad Imam Khomeini assesses each individual crisis situation and then provides what is needed to the best of their ability. Specifically for orphans, Committee Emdad Imam Khomeini has an 'OrphansRespect Plan':

"...orphans are protected by someone who is wealthy...the wealthy person pays a monthly amount into the orphans account...this financial assistance is conveyed to the orphans or their families through Committee Emdad..." (Interview 3)

"People who use this service are under the care of Committee Emdad. People who don't use this service are under the care of Behzisti of the whole country." (Interview 6)

### Non-Governmental Organisations (NGOs)

The support and/or care provided by NGOs takes several forms, but it is often not exclusive to separated and unaccompanied children. Where this is the case, children are not necessarily in their circumstance as a result of a disaster:

### Residential care in the form of small group homes

"...everything is like a home here, just like at home children go out to school...go out to the doctors...we don't even have a sign up outside our organisation as homes don't have signs up outside so we don't have one either ..." (Interview 9)

### Financial assistance and provision of furniture

"...all our children have mothers...we give the families a set amount every month...we check how they use it...we assess what equipment they need and try to provide it...and check that it is still there on a regular basis..." (Interview 20)

"...if a child was cared for by their aunt, in case this aunt didn't have home appliances, we gave them an oven, a fridge...to prevent them from having problems with caring for the child..." (Interview 11)

"...following the Bam earthquake, we support 40 children financially...giving them a monthly allowance..." (Interview 10)

### Arrangement of educational and extra-curricular activities

"Most of our work is teaching children activities of daily living. Also we do some exciting games and we teach painting." (Interview 25)

"...part of our target group also is separated and unaccompanied children...we have some workshops like music classes, computers..." (Interview 28)

### Psychosocial support

"...PTSD [Post Traumatic Stress Disorder] techniques were within the framework of play...games included playing the life train...within this we familiarised children with death, at some stations people got off the train, representing death, at other stops people got on, representing birth..." (Interview 24)

"...all our children have social problems at first, all our carers are trained to deal with these problems and make sure the children are happy..." (Interview 9)

### Provision of emergency relief supplies

"...we usually participate in natural disasters by providing aid and equipment such as blankets, food..." (Interview 10)

### Family Reunification Project

The overwhelming nature of the Bam earthquake lead UNICEF and Behzisti in Kerman to form a joint partnership called the Family Reunification Project, between December 29th 2003 and December 26th 2005, to help reunify families who had become separated as a result of the disaster.

"...the main objective of this agreement being to organise and follow up issues related to children under 18 years of age who have lost their families and parents...." (Interview 23)

Due to the success of the project, it is now continuing with the support of Behzisti:

"...we learned a lot from our experience with UNICEF during this time...and now having seen our results, Behzisti is supporting our project..." (Interview 23)

### Management of Separated and Unaccompanied Children

#### Reasons for Separation

The scale of the disaster provoked an excessive number of agencies to come to Bam, making the area chaotic and meaning that work was uncoordinated, especially as there was no clear organisation in charge:

"...people came to help and to do good, but they made the situation even worse. Some of the children got lost...In English I think you call it lack of management..." (Interview 6)

In the initial stages following the disaster many children were moved out of Bam and cared for elsewhere:

"Following the disaster in Bam, many children were moved out of the city to Mashad, Tehran, Kerman. Very quickly." (Interview 12)

The Family reunification Project could not be started immediately:

"They [separations] could have been avoided but they weren't, and it's partly because UNICEF didn't start family reunification as an immediate programme." (Interview 16)

#### Limiting Separations

No evidence was found of any system in place to limit children becoming separated from their families, before the disaster occurred.

#### Location of Separations

No specific locations of significant numbers of separations were identified.

#### Identification, Registration and Documentation

No clear method by which to identify separated and unaccompanied children was recalled, a variety of responses were given:

"...children were there, just standing on the rubble..." (Interview 6)

"Social workers from Behzisti are supposed to do that [identify unaccompanied children]..." (Interview 16)

Once the registration process was underway, details of separated and unaccompanied children were thoroughly recorded:

"At the time when we find children like this we immediately take their photo with their original clothing, and with any accessories they have with them such as combs or dummies." (Interview 17)

Files relating to unaccompanied children were kept, but the exact content of the folders could not be described:

"All the information relating to the child is documented in their folder; we [Behzisti Bam] have made a database of this information with the help of UNICEF. So we can now access information, like the whereabouts of these children with ease. " (Interview 17)

#### Tracing

A clear procedure for tracing families was not set out in the initial stages of managing the disaster, although later door to door inquiries were conducted:

"... in the emergency phase it's the hospitals, it's the community you always have to talk to, the old people in the community who are usually taking care of these things.." (Interview 16)

#### Reunification

Many reunifications were informal, however the official procedure was described by UNICEF:

"...there is a...set of forms, where it is called closure and reunification, if you find the child and family eligible for reunification this form is filled out and the child and family are taken to a committee called the child welfare committee... the committee consists of the social worker, the manager from Behzisti, one UNICEF staff, then you decide whether, the child can be reunified or not... everything has to be legalised according to the Iranian rules...the department of justice in Bam, has to sign everything and stamp..." (Interview 16)

#### Interim Care

There was definite confusion over interim care in the early stages of the management process. Initially children were taken out of Bam:

"... we took care of many children in Tehran...once the children's families were found, they were returned to their families, those whose families could not be found were taken to Kerman." (Interview 13)

Later, the aim was to first locate the unaccompanied children's extended family members, and then only when this was not an option, to place children in institutional care as close to their home town as possible.

"...previously they [unaccompanied children] were transferred to maintenance centres under the care of the welfare organisation, but this was changed to a better idea; searching for other another relative who can be a guardian for the child..." (Interview 23)

### Durable Arrangements

#### Extended Family

Care within extended family was always the preferred option, and even more so in an extremely family orientated community such as Bam:

"...we formed a relationship with extended family members of unaccompanied children, and tried to help them look after orphaned children from their own family, helping them financially or with things like furniture, but if they still couldn't look after the child, we tried a different family member, only if there was no-one left, and as a last choice did we take these children to our [Behzisti's] own facilities..." (Interview 23)

#### Community Care

Community care does not exist in Iran, neither does foster care in the British form, due to religious and cultural reasons:

"...Because fostering with someone who is not related to you by blood, if the child is above six or seven, then the child would not be halal, if it's a boy to the mother and vice versa." (Interview 16)

#### Institutional Care

Only when extended family could not care for the unaccompanied child, even with support from other agencies, was institutional care chosen in the form of group homes:

"orphanages are no longer in use in Iran, we realised they had a negative impact. Now we have Children and Young Adult Houses, these houses have the aim of making the environment as similar to a family home as possible. Before 50 - 70 children would be housed in one place, and now the maximum number is 20." (Interview 15)

#### Adoption

No international adoption was reported following the earthquake, although national adoption did take place:

"...if the child does not have anyone upon agreement of the General Prosecutor [member of the Iranian judicial system] we allow other people to adopt these children..." (Interview 13)

Behzisti cannot allow children to be adopted without permission from the Iranian courts. This permission is given under specific conditions that include the following:

"...the family must not have any children of their own...have been married for five years and not have been able to get pregnant during this time, this must also be certified by a doctor, they must be the appropriate age for adoption...people who are wealthier and more educated are prioritised..." (Interview 13)

"...court states that one third of the new families legal possessions should be put in the child's name. Once the court has given permission, the child is still under the supervision of social workers in the their new family, until they have been with the family for a period of six months, at which point the court issues a letter allowing the child's new family to obtain a birth certificate for them." (Interview 17)

### Non-Governmental Organisations in Bam

In general the role of local, national and some international NGOs in Bam was, and still is, to support unaccompanied children. In contrast to the findings in Part one, relating to Iran in general, it was not deemed suitable following the earthquake to delegate the responsibility of permanently caring for unaccompanied children to NGOs. However, the rest of their roles were similar to the roles of NGOs described in part one of this study:

"...we seek support from other NGOs... we allow them to work with us. Behzisti does not give children to NGOs, for example there is a NGO run play school for children, but these children only visit this NGO centre during the day, they do not stay the night as they go back to their families in the evening." (Interview 17)

## Discussion

### The Role of NGOs in the Management of Separated and Unaccompanied Children, following Disasters in Iran

Research has shown that NGOs in Iran have a restricted role in the management of separated and unaccompanied children. Firstly, it should be noted that NGOs are viewed with some scepticism in Iran. Even though the number of NGOs has been increasing since1990, Iran is still unfamiliar with their work.

Literature obtained at the Educational Research Centre of Behzisti in Tehran, acknowledged that the general consensus in Iran is that NGOs should be "managed under the supervision of, and their programs should be coordinated by, governmental organisations", however cooperation between agencies was stressed [18]. This questions whether NGOs actually exist in Iran, as they largely do not conform to the standard definition.

As a consequence of the way in which NGOs are viewed in Iran, combined with the fact that separated and unaccompanied children are extremely vulnerable, it is not surprising that few NGOs in Iran are delegated the responsibility of caring for unaccompanied children. Yet the fact that in general NGOs play at least a supporting role in the care of separated and unaccompanied children, shows that progress is being made.

In addition, the majority of NGOs interviewed as part of this research have multiple roles, one of which involves separated and unaccompanied children. Few institutes cater exclusively for unaccompanied children, and no organisations were found to deal solely with children separated or unaccompanied as a result of disasters. This may be due to the fact that NGOs are limited in the care they can provide, as the majority of the responsibility for separated and unaccompanied children lies with Behzisti (the government welfare organisation of Iran). Alternatively organisations may have chosen not to segregate unaccompanied children to avoid children being stigmatisatised. Research has also shown that community involvement in NGO lead projects is high, and as a result many projects are proving successful.

Furthermore, as the work of NGOs in Iran is still relatively new, especially in rural areas, their work is yet to take an established form. There is also little cooperation between NGOs, especially between local and international organisations. In spite of this, coordination between government and international agencies seems to be improving, especially after the success of recent collaborations such as the Family Reunification Project.

### The Role of other agencies in the Management of Separated and Unaccompanied Children, following Disasters in Iran

Research has shown that the work of a number of other agencies, actively involved in the management of separated and unaccompanied children in Iran, is related to the work of NGOs.

### Iranian Red Crescent

The Iranian Red Crescent organisation, a member the world's largest humanitarian network [19], was found to be central in providing basic relief supplies [4,20,21] to all victims of disasters, immediately after the event.

### Behzisti

Management of separated and unaccompanied children following disasters is only a small part of Behzisti's work, as it has a range of duties to uphold in Iran. Behzisti outlined a substantial number of conditions NGOs must follow in order to be allocated the duty of caring for unaccompanied children. The organisation conveyed a firm control over NGO activities.

### Committee Emdad Imam

Committee Emdad Imam Khomeini has a significant place within Iranian society. In contrast, some independent NGOs are yet to gain the credibility and recognition they deserve. Committee Emdad Imam Khomeini has various roles, but these are not distinct, and in spite of its good work many of its activities seem to overlap with the work of other agencies. Schemes similar to the Orphans Respect Plan are common in Iran and not just for orphans but also for families who struggle financially. A great deal of financial assistance is given in Iran and other forms of support, including psychosocial, are on the increase.

### United Nations

United Nations agencies, such as WHO and UNICEF, are continuing to develop their relationship with Iran. For example, following the Bam earthquake time was needed to gain permission for projects, but once consent was given cooperation was commendable. As trust is developed, progress should occur at a more rapid pace.

### International Guidelines on the Management of Separated and Unaccompanied Children

Only UNICEF referred to the guidelines for the management of Separated and Unaccompanied children developed by the inter-agency working group [22]. This was unexpected as the literature review indicated these universal guidelines were widely used. Other organisations made vague references to other documents, however there was no evidence suggesting a definite set of guidelines were in active use throughout Iran. Even though documentation was only rarely referred to, many of the principles contained within the international guidelines on the management of separated and unaccompanied children were put into practice in Bam. This was especially true once UNICEF's assistance began in accordance with international guidelines. These state that when an event overwhelms the national government, the victims of the disaster are the responsibility of the United Nations [4], after which other humanitarian organisations should assist where appropriate.

### Reasons for Separation

As stated in the literature on this topic, children were separated from their families as a result of the way in which organisations initially worked [7,5,23] following the disaster in Bam. However, neither the scale of the disaster nor the necessity of UNICEF's input could have been predicted. Furthermore, the choice of residential care options was not limited to begin with as recommended [23,24], leading to a number of separations occurring immediately after the earthquake.

### Limiting Separation

No evidence of implementing recommendations to limit family separations [8,22,23,25] following the Bam earthquake was found. Although after the subsequent earthquake in Zarand (a city in the same province), the Family Reunification Team that worked in Bam assisted Behzisti in Zarand. Even so, a preparedness plan is yet to be developed and implemented.

### Location of Separations

As no specific locations of significant numbers of separations were identified, suggestions relating to methods of reducing separations 5 were not followed.

### Identification, Registration and Documentation

Guidelines [5,7,23,26,27] did not describe the process via which children could be identified, and at operational levels there was no clear system. Where possible, children were sensitively interviewed and registered systematically in accordance with international guidelines [5,23,26]. Documentation was kept confidential, and steps were being taken to make information storage standardised through the use of a database.

### Tracing

Tracing family members or primary legal or customary care-givers was done by actively searching as suggested in the guidelines [5]. However, computerised cross-matching was not used.

### Reunification

Formal reunification procedures followed the guidelines [23]; the validity of the relationship between those being reunited was demonstrated by the Child Welfare Committee and in some cases ongoing support was also provided following reunification [1].

### Interim Care

After the initial chaos following the Bam earthquake, guidelines [5,7] were followed in that unaccompanied children were cared for by relatives and only placed in residential care when this was not possible.

### Durable Arrangements

In accordance with international guidelines [5,23] durable arrangements that would hinder eventual family reunification were not made until tracing efforts had been exhausted.

### Extended Family

In line with the guidelines [5,7,28] kinship care was always the preferred option for the people of Bam, as well as those coordinating reunification efforts.

### Community Care

Again guidelines [23] were adhered to when community and foster care were not arranged, in line with the Islamic Shaaria law in Iran.

### Institutional Care

As suggested [7,23] institutional care was only used as a last resort, and such care has been improving in Iran recently with the formation of small group homes.

### Adoption

Following international guidelines, unaccompanied children were not adopted until their status had been clarified [27-29]. The country's rules were considered, along with the circumstances of the situation, from which inter-country adoption was deemed inappropriate.

Overall systems for managing separated and unaccompanied children following disasters in Iran, involving NGOs are emerging. However, most are yet to be formalised.

### Recommendations for Future Management

#### Coordination between Agencies

Methods to improve coordination between agencies, following a disaster in Iran, include designating a named person or team to act as a crisis manager, clearly defining the role of each agency involved, and emphasising teamwork. Cooperation between local and international agencies in Iran should be the focus of improvement. The existence of the UN Office for the Coordination of Humanitarian Affairs (OCHA) [30] was only mentioned during a UNICEF interview, therefore OCHA needs to further publicise it's capabilities if it is to play a more active role in disaster management in Iran.

#### Preparedness Planning Guidelines

Personnel involved in disaster management should be encouraged to record their experiences so that empirical evidence can be combined with theoretical knowledge. Subsequently informed guidelines and preparedness plans, to manage separated and unaccompanied children following disasters, can be developed and localised.

#### Training

Personnel involved in the management of separated and unaccompanied children in Bam helped Behzisti in Zarand following the earthquake there. However, efficient training on how to manage separated and unaccompanied children following a disaster, before another event, would be more cost and time effective in the long term. Training should involve numerous well respected community members, to help empower the community and to ensure there is always at least one trained person available.

#### Raising Awareness

Raising awareness of the specific dangers to unaccompanied children following a disaster, appropriate methods of management and the services available to the public, would give Iranian citizens the knowledge to work together to prevent unaccompanied children suffering unnecessary distress.

### Limitations

#### Methodological Limitations

To fulfil the Principal Investigator's International Health BSc requirements, the study could only be 6 weeks long, and involve 25 meetings and interviews, in two regions of Iran. Children were not interviewed as this was not deemed ethical.

Data is only from two areas of Iran, and direct observation in the field was not possible as this is a retrospective study. It is therefore contextualised in these settings at the time of the study, and limited to the experiences of the individual participants. Thus it may not be generalised to every disaster that has occurred in Iran. This issue has been addressed by clearly stating when data only relates to a specific region.

A comprehensive list of NGOs responsible for managing separated and unaccompanied children, following disasters in Iran is not available; therefore a convenience sample had to be recruited.

Subjects introduced the Principal Investigator to contacts they deemed suitable. Even though this was useful and expanded the study it may have lead to only selective people being interviewed.

The use of a translator was a limitation. Although the translator was debriefed beforehand to encourage a direct translation and use of a translator was kept to a minimum, as the Principal Investigator speaks fluent Farsi. Using a translator also allowed subjects to speak in the language they felt most confident in.

Interviews acquired via email are not as reliable as primary evidence, although measures were taken to validate information gathered via this method.

As a female student who studies in the UK, the interviewer may have unintentionally influenced the subject's responses.

#### Limitations of Data Collection and Analysis

Background noise may have affected the quality of some recordings. Participants may not have been entirely frank due to researcher unfamiliarity and/or perceived negative impacts for themselves or the organisation they are involved with, despite emphasis on the fact that there would be no negative repercussions from participating in the study.

This study was done approximately two years and five months after the Bam earthquake in 2003, in which time subjects have had the opportunity to reflect on their experiences. However, the research was carried out in the subjects own environment so they were not removed from the disaster site.

## Conclusion

Research has shown that NGOs do not have a leading role in the management of separated and unaccompanied children, following disasters in Iran. Behzisti, the Iranian government's welfare organisation, is mainly responsible for these children and is aided by United Nations agencies, such as UNICEF, in crisis situations. Committee Emdad Imam Khomeini is prominent in Iran as it is under the direct supervision of the leading power in the country. It's principal aim is to provide assistance to those who are disadvantaged, such as separated and unaccompanied children. Largely NGOs provide financial assistance, furniture, psychosocial support, emergency relief supplies and arrange educational and extra-curricular activities for unaccompanied children in Iran. However, only in rare cases are these provisions solely for children unaccompanied as a result of a disaster, most are for children unaccompanied due to a variety of reasons including abandonment and divorce.

This study allowed the development of recommendations to improve the management of separated and unaccompanied children, following disasters in Iran. These recommendations include greater co-ordination between agencies involved in disaster management. Preparedness planning, which involves documentation of experiences to develop protocols, as well as training and raising awareness relating to disaster management. Even though there is a lack of formal guidelines regarding separated and unaccompanied children in Iran, the majority of the international recommendations are being followed. This means that children are being identified as promptly as possible following a disaster, then and their families traced. Once tracing efforts have been exhausted, other favourable options such as care within extended family or the community are investigated, before deciding on the less favourable option of institutional care. If cooperation between the government, international, national and local agencies continues, the emerging systems will be formalised with ease.

## Competing interests

The authors declare that they have no competing interests.

## Authors' contributions

FB conceived the study, participated in its design, co-ordination and data collection, and drafted the manuscript. HRB participated in the co-ordination and helped draft the manuscript. Both authors read and approved the final manuscript.
